# Prediction models for *Mtb* infection among adolescent and adult household contacts in high tuberculosis incidence settings

**DOI:** 10.1371/journal.pgph.0004340

**Published:** 2025-03-31

**Authors:** Edson Tawanda Marambire, Claire J. Calderwood, Leyla Larsson, Kathrin Held, Palwasha Khan, Denise Banze, Celina Nhamuave, Lillian T. Minja, Alfred Mfinanga, Rishi K. Gupta, Celso Khosa, Junior Mutsvangwa, Norbert Heinrich, Katharina Kranzer

**Affiliations:** 1 The Health Research Unit Zimbabwe, Biomedical Research and Training Institute, Harare, Zimbabwe; 2 CIH^**LMU**^ Center for International Health, University Hospital, LMU Munich, Germany; 3 Faculty of Infectious and Tropical Diseases, London School of Hygiene & Tropical Medicine, London, United Kingdom; 4 Institute of Infectious Diseases and Tropical Medicine, LMU University Hospital, LMU Munich, Munich, Germany; 5 Instituto Nacional de Saúde, Marracuene, Mozambique; 6 National Institute for Medical Research - Mbeya Medical Research Centre, Mbeya, Tanzania; 7 University College London, London, United Kingdom; 8 Biomedical Research and Training Institute, Harare, Zimbabwe; 9 German Center for Infection Research (DZIF), Partner Site Munich, Munich, Germany; 10 Fraunhofer Institute for Translational Medicine and Pharmacology ITMP, Immunology, Infection and Pandemic Research, Munich, Germany; Emory University Rollins School of Public Health, UNITED STATES OF AMERICA

## Abstract

Tuberculosis household contacts are at high risk of developing tuberculosis. Tuberculosis preventive therapy (TPT) is highly effective, but implementation is hindered by limited accessibility of diagnostic tests aimed at detecting *Mycobacterium tuberculosis* (*Mtb)* infection. Development of *Mtb* infection prediction models to guide clinical decision-making aims to overcome these challenges. We used data from 1905 tuberculosis household contacts (age ≥10 years) from Zimbabwe, Mozambique and Tanzania to develop two prediction models for *Mtb* infection determined by interferon-gamma release assay (IGRA) using logistic regression with backward elimination and cross-validation and converted these into a risk score. Model performance was assessed using area under the receiver operating characteristic curve (AUROC), sensitivity, and specificity. We developed a basic model with six predictors (age, caregiver role, index case symptom duration, index HIV status, household crowding, and index GeneXpert MTB/Rif results) and a comprehensive model with eleven predictors. The basic and comprehensive risk scores showed limited predictive capability (AUROC 0.592, sensitivity 76%, specificity 35% and AUROC 0.586, sensitivity 76%, specificity 36% respectively), with considerable overlap across IGRA-positive and -negative individuals. Neither model conferred net benefit over a treat-all strategy. Overall, our results suggest that the prediction models developed in this study do not add value for guiding TPT use in high-tuberculosis burden settings. This likely reflects complex *Mtb* transmission dynamics at the household- and community-level, variation in individual-level susceptibility and immune response, as well as limited accuracy of IGRA testing. Improved diagnostics to determine *Mtb* infection status in terms of ease-of-use, accuracy, and costs are needed.

## Introduction

Whilst in 2020 COVID-19 briefly overtook tuberculosis (TB) as the leading cause of death due to an infectious disease, TB has since regained its lead, despite being both preventable and curable [1]. TB is caused by pathogens of the *Mycobacterium tuberculosis* (*Mtb*) complex and is transmitted primarily by aerosols, putting people living with someone who has TB at particularly high risk of *Mtb* infection [2].

It is estimated that a quarter of the world’s population is infected with *Mtb*, representing a vast reservoir of people at risk of developing TB [3]. One in ten people infected with *Mtb* will progress to TB [4–6], risk of progression can be significantly reduced (≤90%) by preventive therapy (TPT) which is an important pillar in the WHO End-TB strategy [7,8]. Identifying those with *Mtb* infection is a critical step in the TB prevention cascade, especially in high-burden settings. Household contacts of individuals recently diagnosed with TB represent a key population for screening and preventive interventions because of their high likelihood of exposure to *Mtb* [9,10].

The most recent WHO guidelines recommend expanding TPT to all household contacts in high-TB incidence countries [11], if possible focused on those with demonstrated *Mtb* infection, but few countries have operationalised this. Diagnostic tests to detect *Mtb* infection include tuberculin skin tests (TST) and interferon-gamma release assays (IGRA). These tests require nursing skills for intradermal injections, established cold chain, and repeat patient visits or trained laboratory staff and infrastructure. A lack of cheap, rapid and easy to use diagnostic tests to screen for *Mtb* infection partly explains the suboptimal implementation of TPT for TB household contacts [12,13].

Given costs and logistics of these tests, most high-TB burden countries either i) recommend TPT for all household contacts without prior testing for *Mtb* infection, resulting in considerable overtreatment and health-system costs [14], or ii) restrict TPT to high risk groups (such as household contacts less than 5 years old and people living with HIV).

Considering these challenges, recent efforts have focused on improving strategies to identify *Mtb* infection among household contacts, including use of prediction models based on easily ascertainable risk factors [15]. These models aim to incorporate individual- and household-level, environmental, host- and pathogen-related predictors such as proximity to the person with TB, time spent with the index case, and the infectiousness of the person with TB [16–19]. Ideally, the predictors would be easy to ascertain from the person diagnosed and being treated for TB by a nurse or community health worker, thus reducing the burden on health systems by prioritizing individuals or households for individual assessment, diagnostic testing and subsequent preventive therapy where indicated. Previous models have shown inconsistent performance, with some achieving moderate predictive accuracy in low-TB burden settings but often underperforming in high-TB burden settings [16–19], highlighting the challenges of generalizability across diverse populations and epidemiological contexts.

Here, we sought to develop and systematically evaluate whether prediction models can accurately predict IGRA positivity as a marker of *Mtb* infection among adolescent and adult TB household contacts in East and Southern Africa, to support targeted provision of TPT without IGRA or TST.

## Methods

In a dataset of adult and adolescent TB household contacts from Tanzania, Mozambique, and Zimbabwe, we developed *de novo* prediction models for positive IGRA result using a large and diverse cohort with information related to household member with TB (index case), household, and household contact factors, measured at the time of diagnosis of the index case in the Early Risk Assessment in TB contactS by new diagnostic tEsts (ERASE-TB) study. Transparent Reporting of a Multivariable Prediction Model or Individual Prognosis Or Diagnosis (TRIPOD) guidelines are used for reporting of results [20].

### Study population

**Fig 1:**
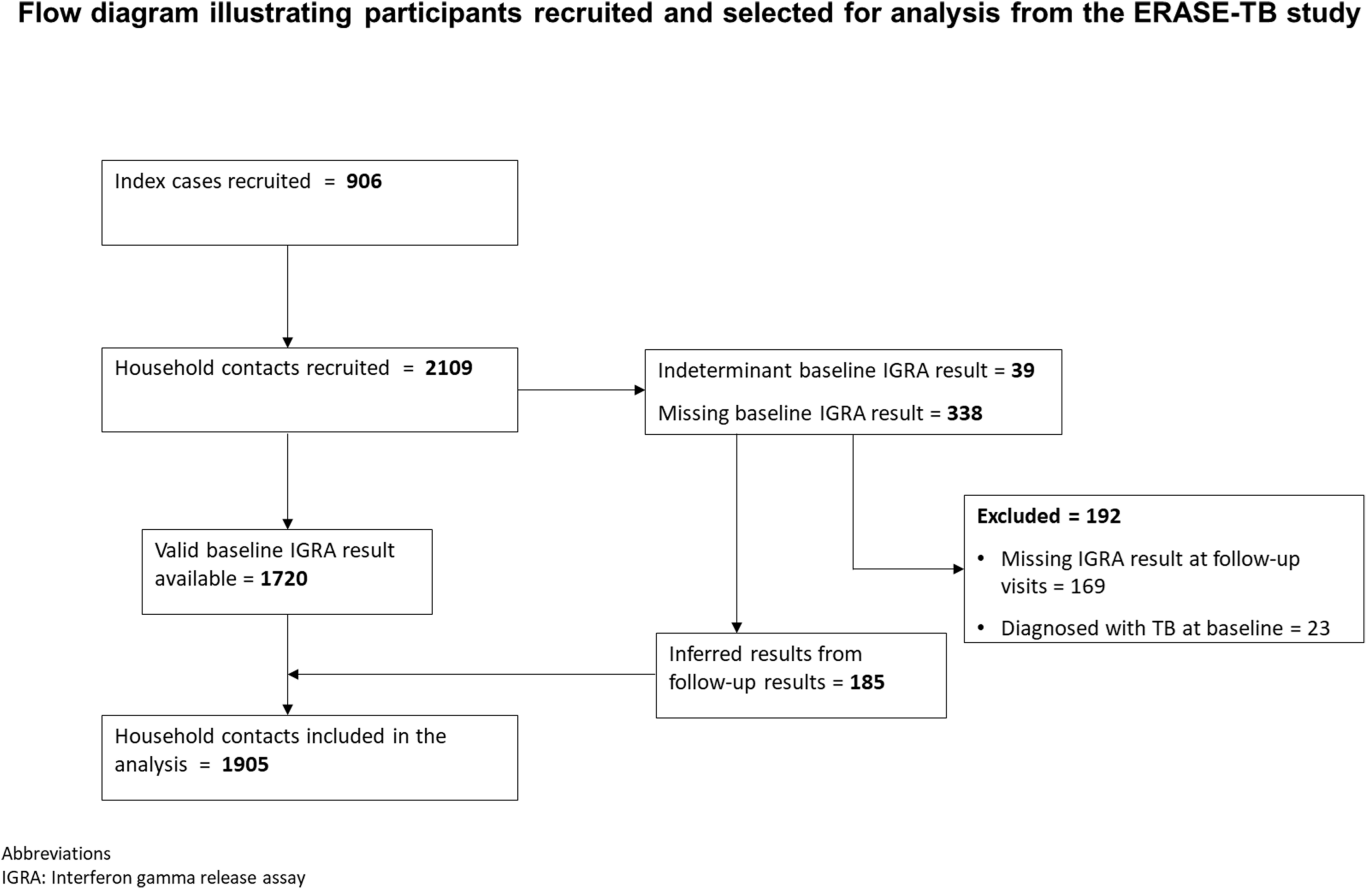
Flow diagram illustrating participants recruited and selected for analysis from the ERASE-TB study.

The study population for the development of prediction models was a large multi-national, observational, prospective cohort study (ERASE-TB). Inclusion and exclusion criteria are shown in S1 Table. We enrolled household contacts (age ≥10 years) of adults with microbiologically confirmed pulmonary TB (age ≥18 years) with a positive smear (at least +1) and/or a medium or high-level positive Xpert MTB/Rif sputum result across three sites: Maputo (Mozambique), Mbeya (Tanzania), and Harare (Zimbabwe). The enrolment periods were as follows: Maputo from 05 August 2021 to 23 March 2023, Mbeya from 31 August 2021 to 08 February 2023, and Harare from 08 March 2021 to 17 January 2023. A total of 2109 household contacts were recruited (Fig 1).

### Study procedures

The study protocol for ERASE-TB, including details of all study procedures, has been published [21]. In brief, at each study visit (every 6 months for a period of up to 24 months), enrolled household contacts underwent TB screening (WHO symptom screen and chest X-ray, followed by Xpert MTB/Rif Ultra if either was suggestive of TB) and collection of samples for novel diagnostic tests and biobanking. All household contacts who were not known to be living with HIV were offered HIV testing. IGRAs (STANDARD^TM^ F TB-Feron FIA [IFN-gamma; SD Biosensor, Republic of Korea]) were conducted at baseline and at subsequent visits dependent on reagent availability. Individual- and household-level questionnaires collected socioeconomic characteristics, information about living conditions, past medical history, and factors known to be associated with *Mtb* infection.

### Outcome

The primary outcome of interest was IGRA binary result (negative or positive) as a marker of *Mtb* infection, interpreted according to the manufacturer’s instructions. For household contacts with indeterminant or missing IGRA results at baseline, the next available follow-up IGRA result was used to infer baseline results: a negative IGRA result at follow-up was categorised as negative, and a positive IGRA result at follow-up was categorised as positive. Household contacts diagnosed with TB within 30 days of the baseline visit and those with missing or indeterminate IGRA result after taking into account follow-up results were excluded from the analysis.

### Development of prediction models

Analysis was done using R version 4.3.2. Inclusion of candidate predictor variables for the newly developed model was based on existing evidence of association with *Mtb* infection, according to reviewed literature [19,22–31] and availability in the ERASE-TB dataset. Variables related to the person diagnosed and treated for TB were regarded as household-level variables. Prior to model development, descriptive analyses were conducted. Continuous data were summarized by the mean and standard deviation or median and interquartile ranges, while categorical data was summarized as frequencies with percentages. For model development, the ERASE-TB dataset was randomly split into model development (70%) and evaluation (30%) datasets. Missing values in predictor variables were classified as “unknown”.

We utilized 10-fold cross-validation technique exclusively on the training data. This technique involved dividing the training set into 10 equal subsets, iteratively training the model on 9 of these subsets, and validating it on the remaining subset. This process was repeated 10 times, ensuring that each subset served as the validation set exactly once. Performance metrics obtained from each fold were averaged to provide a robust estimate of the model’s performance.

Two models were developed. We first developed a model with basic individual- and household-level predictors, ascertainable from the person with TB by a nurse or community health worker using just a questionnaire (basic model). Our goal was to be able to predict which household contacts had *Mtb* infection without having to physically review and assess every individual member of the household. We secondly developed a model with all eligible individual and household predictors (comprehensive model), to determine whether predictive performance could be enhanced using a more comprehensive approach. S2 Table shows candidate predictor variables and how data was collected.

For each model, using the training dataset, we conducted a multiple logistic regression, with backward elimination of candidate predictors contributing little predictive value to the overall multivariable model, assessed using the “varImp” function in R and examining regression coefficients and p-values (i.e., sequential removal of variables with the largest p-value and least important until variables with p-value < 0.2 remained). To derive a prediction risk score to be used by clinicians in resource-limited settings, regression coefficients were normalized into scores using the formula:


$$Score=\frac{Coefficient-Lowestcoefficient}{\text{Highest coefficient}-Lowestcoefficient}\times 10$$

Scores ranged from 0 to 10 (for category with the lowest and category with the highest predictive impact respectively) and were added up to compute a summative risk score for *Mtb* infection.

### Risk score assessment

We assessed the predictive performance of our de novo basic and comprehensive risk scores by evaluating the agreement between observed and predicted risks using the held-back evaluation dataset. A confusion matrix was then used to assess the model’s sensitivity and specificity, with a decision threshold of 0.5 and the corresponding maximum Youden Index, which indicates the optimal balance between sensitivity and specificity, was calculated. We examined area under the receiver operating characteristic (ROC) curves to compare the performance of the two risk scores and determine the added value of including detailed clinical information in the model. We stratified individuals into low-, medium-, and high-risk groups for *Mtb* infection based on score categories.

We also visually displayed the relationship between the scores and the outcome using density plots, to understand how well the scores correlated with the outcome measurement. Decision curve analysis was used to compare the net benefit of interventions to treat *Mtb* infection based on risk scores against treat-all or treat-none approaches across different threshold probabilities.

To evaluate the robustness of the model, a sensitivity analysis was conducted using the basic model. This analysis included only participants with baseline IGRA results, excluding inferred results. Additionally, the results from the last available IGRA test during follow-ups were utilized.

### Ethical considerations

Informed written consent, or guardian consent and individual assent for people under the age of 18, was obtained from all participants. Ethical approval was granted by all relevant institutions: the Medical Research Council in Zimbabwe (MRCZ/A/2618), the National Health Research Ethics Committee in Tanzania (TMDA-WEB0021/CTR/0004/03), the National Bioethics Committee for Health in Mozambique (541/CNBS/21), and the ethical committees of London School of Hygiene & Tropical Medicine, United Kingdom (22522–2) and the medical faculty of the Ludwig-Maximilians-Universität München, Germany (20–0771)).

## Results

### Study population

In ERASE-TB, 2109 household contacts of people diagnosed with pulmonary TB were recruited from 822 households between March 2021, and March 2023. A total of 1905 (90%) were included in the analysis. Of these, 1720 (90%) had a valid baseline IGRA result and an additional185 (10%) had results inferred from follow-up results (Fig 1). 169 (8%) participants could not be classified into the outcome of interest even after inferring follow-up results and 23 (1.1%) were diagnosed with TB at baseline, and thus were excluded from the analysis. Therefore, 1905 (90% recruited) household contacts were included in the analysis. Table 1 describes the baseline individual- and household-level characteristics of included participants: 62% were female and median age was 27 (interquartile range: 17-42) years with 29% of participants being adolescents (10-17 years). A total of 292 (15%) household contacts were living with HIV. Overall, 811/1905 (43%) of the included participants were IGRA positive. The highest prevalence of IGRA positivity was in Zimbabwe (49%) and among older adults (50%, compared to 33% among adolescents aged 10-14 years).

**Table 1:** Baseline characteristics of participants.

		IGRA-Positive	IGRA-Negative
		N=811 (43%)	N=1094 (57%)
		N (%)	N (%)
INDIVIDUAL LEVEL CHARACTERISTICS (HOUSEHOLD CONTACT)			
Country	Zimbabwe	332 (49%)	342 (51%)
	Mozambique	197 (32%)	411 (68%)
	Tanzania	282 (45%)	341 (55%)
Age (years)	10-17	197 (35%)	358 (65%)
	18-25	149 (40%)	226 (60%)
	26-35	124 (42%)	172 (58%)
	36^+^	341 (50%)	338 (50%)
Sex	Female	511 (43%)	677 (57%)
	Male	300 (42%)	417 (58%)
HIV status of household contact	HIV negative	693 (44%)	895 (56%)
	HIV positive	112 (38%)	180 (62%)
	HIV status unknown	6 (24%)	19 (76%)
BMI category^*^	Underweight	72 (43%)	96 (57%)
	Normal weight	490 (41%)	697 (59%)
	Overweight	155 (44%)	197 (56%)
	Obesity	94 (48%)	104 (52%)
Frequency of contact with index person with TB	<1 day per week	9 (53%)	8 (47%)
	1-3 days per week	26 (43%)	34 (57%)
	4-6 days per week	38 (44%)	49 (56%)
	Daily	737 (42%)	1002 (58%)
Directly involved in caring for the index person with TB	No	437 (39%)	675 (61%)
	Yes	374 (47%)	419 (53%)
HOUSEHOLD LEVEL CHARACTERISTICS			
Household income per person per day (USD)	Median (IQR)	0.55 (0.30–1.06)	0.55 (0.31–0.98)
Impoverishment (< 1.90 USD/day)	Yes	751 (42%)	1030 (58%)
	No	60 (48%)	64 (52%)
Xpert semi-quantitative grade of TB index case	High	499 (44%)	632 (56%)
	Medium	312 (40%)	462 (60%)
HIV status of index	Unknown	68 (46%)	80 (54%)
	Negative	533 (45%)	665 (55%)
	Positive	210 (38%)	349 (62%)
Index ART status^**^	Not on ART	48 (29%)	118 (71%)
	On ART	162 (41%)	231 (59%)
Index TB presenting symptoms	Cough	808 (43%)	1089 (57%)
	Haemoptysis	163 (43%)	217 (57%)
Duration of index case symptoms before start of treatment (days)	<30 days	91 (31%)	200 (69%)
	30-89 days	348 (41%)	502 (59%)
	≥90 days	372 (49%)	387 (51%)
Crowding^***^	No	619 (41%)	876 (59%)
	Yes	192 (47%)	218 (53%)

Footnotes:

BMI = body mass index.

IGRA = interferon-gamma release assay.

TB = tuberculosis.

IQR = inter-quartile range.

ART = anti-retroviral therapy.

UN = United Nations.

USD = United States Dollars

* BMI Categories were created using the WHO categories [32]

** ART status among index cases with HIV only

*** Crowding categorised as per UN Habitat definitions (≥3 people per room) [33].

### Development and validation of risk prediction model

The training subset consisted of 1334 (70%) household contacts, and evaluation subset 571 (30%). Eleven individual- and household-level *Mtb* candidate predictor variables were included in the initial multivariable logistic regression (S1 Code), constituting the comprehensive model (S3 Table). The final basic model included six individual- and household-level predictors. Individual-level predictors were age, and whether the household contact cared for the index, while household-level predictors included duration of illness of the index case, index HIV status, Xpert/smear positivity level, and household crowing according to United Nations definition (≥3 people per room) (Table 2). TB index cases, not on ART contributed the lowest score while index symptom duration of more than 3 months had the highest score.

**Table 2:** Basic model predictors of *Mtb* infection.

	Coefficient	P-Value	Score
Mtb risk factors			
Age (years)			
10-14			4
15-17	0.2813	0.217	6
18-25	0.1880	0.331	5
26-35	0.3227	0.104	6
36 +	0.6677	<0.001	9
Cared for the index			
No			4
Yes	0.2785	0.021	6
Index HIV status			
Negative			4
Positive, not on ART	-0.4768	0.027	0
Positive, on ART	-0.0623	0.668	3
Unknown	0.3231	0.126	6
Index Symptom duration			
<1 month			4
1≤ months<3	0.5828	0.001	8
3 months and above	0.8447	<0.001	10
Household crowding (≥3 people per room)			
No			4
Yes	0.2629	0.058	6
GeneXpert MTB/Rif Ultra index positivity			
Medium			4
High	0.1801	0.498	5
*Lowest/Highest possible score*			***20/42***

**Fig 2:**
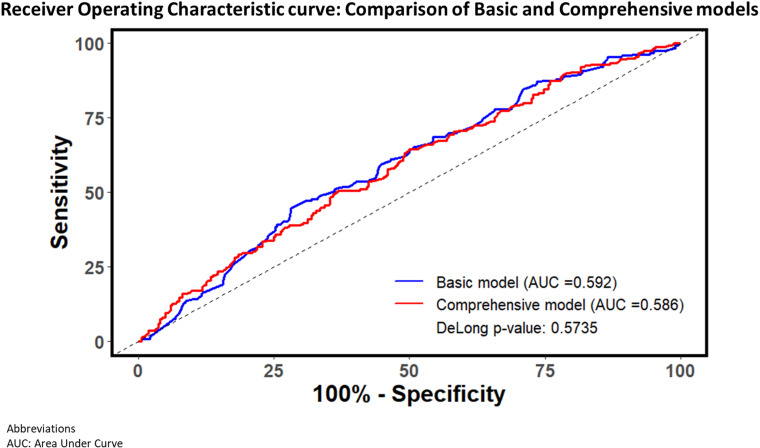
Performance of basic model and comprehensive models.

Neither the basic nor the comprehensive model achieved adequate prediction for positive IGRA result among TB household contacts. The basic model yielded low AUROC (0.592; Fig 2). At a threshold of 0.5, the model had a sensitivity of 76% (95% CI: 70 – 79), specificity of 35% (95% CI: 27 – 39), and Youden Index of 0.08 (95% CI: 0.01 – 0.15). The comprehensive model had an equally low AUROC of 0.586, while at a threshold of 0.5 the model had a sensitivity of 76% (95%CI: 72 – 82), specificity of 36% (95% CI: 30 – 42) and Youden Index of 0.13 (95% CI: 0.05 – 0.19). Sensitivity analysis of the basic model using only participants who had baseline IGRA results available (without inferring missing or indeterminate baseline result) as well as using results of last available IGRA test result during follow-up did not change the model performance, yielding AUROCs of 0.61 and 0.60 respectively (S1 Fig**).**

**Fig 3:**
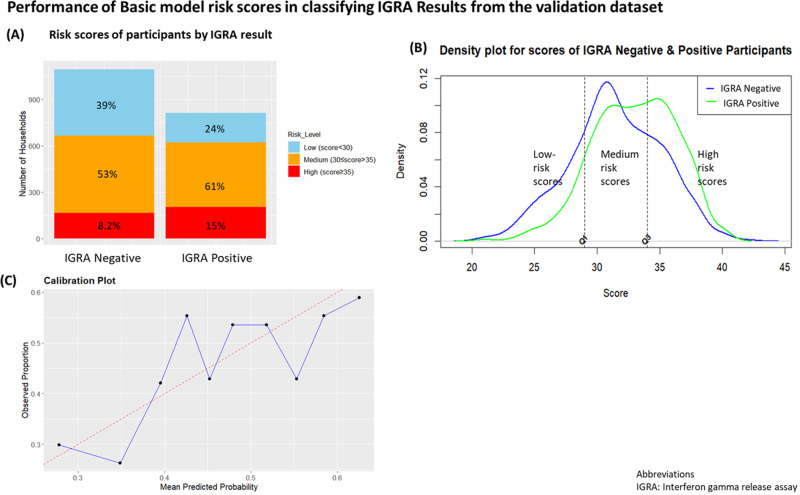
Performance of *de-novo* risk scores on positive and negative IGRAs.

We developed a risk score to assess the risk of positive IGRA result in a clinical setting, based on the basic prediction model (Table 2**).** Each response to a variable in the final model contributed to the score independently. The maximum possible risk score for the model was 42, while the minimum score was 20. Risk score categories were developed as low positive IGRA risk (<30), medium positive IGRA risk (30≤score>35), high positive IGRA risk score (≥35). These categories were determined based on the distribution of the scores, where approximately 25% of the scores are less than 30, and the upper 25% are above 35. This approach was taken to ensure a balanced representation across the risk categories. Risk score categories among IGRA positive and IGRA negative participants showed a small difference in proportions of people categorized as medium- and low-risk in both groups (Fig 3A). Using this risk stratification, the basic model classified 53% and 8.3% of IGRA negative participants as medium and high risk respectively, while 24% of IGRA positive participants were classified as low risk. The density plot revealed substantial overlap between the scores assigned to IGRA negative and positive participants, with the IGRA positive group showing a distribution with slightly higher scores compared to the IGRA negative group (Fig 3B). This overlap implies that the model does not distinguish between these two groups effectively, overestimating risk for IGRA negative contacts, and underestimating risk for IGRA positive contacts. This is further evidenced by the calibration plot (Fig 3C), (**S2** **Code)** which demonstrates that the model’s predicted probabilities do not align well with the observed outcomes.

**Fig 4:**
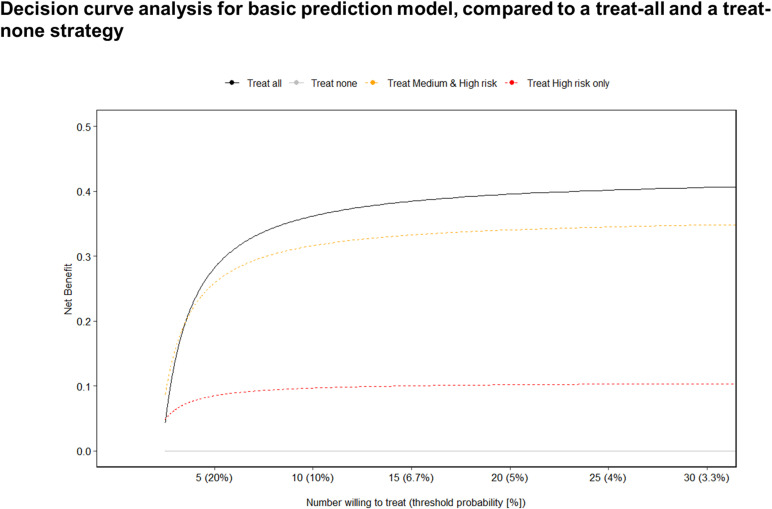
Decision curve analysis for basic prediction model, compared to a treat-all and a treat-none strategy.

Decision Curve Analysis (DCA) of our basic model predicting IGRA positivity compares the net benefit of interventions to treat *Mtb* infection based on risk scores against treat-all or treat-none approaches across different threshold probabilities (i.e., the probability of disease at which the health system/provider would provide treatment). When considering our model in the context of TB preventive interventions for TB household contacts, the net benefit of treat-all decision surpasses that of using risk scores across the range of threshold probabilities considered (Fig 4**).**

## Discussion

TB remains a significant global health challenge, particularly in resource-limited settings where the burden of disease is highest [34]. Using risk prediction tools to guide use of TPT among household contacts has been proposed as one pragmatic approach to improve delivery of TPT in settings where current diagnostic tests are not feasible.

Our findings do not support use of risk prediction tools for *Mtb* infection - as defined herein by IGRA testing – among adolescents and adults in high-TB burden settings. We developed two new tools using high-quality granular data from a research cohort of adolescents and adults from TB-affected households. Both achieved poor predictive accuracy, AUC, and sensitivity and specificity estimates. In short, each model was no better than random selection. For example, our basic risk score inadvertently misclassified 61% of individuals who were not infected with *Mtb* determined by IGRA as being medium- to high-risk. Given the high proportion of household contacts misclassified by these scores, we do not recommend their use in clinical practice.

Our study highlights the challenges of developing effective prediction models for *Mtb* infection in high-TB burden settings. While previous models, such as the Mandalakas score, have shown reasonable performance in specific populations (e.g., children younger than six years), their application to adolescent and adult populations in high-TB burden environments remains problematic [18,19,31]. However, this finding is in contrast with *Mtb* infection prediction models developed in low-TB burden settings that have achieved reasonable predictive performance among adolescents and adults [16,17]. This brings to attention the complexity of *Mtb* transmission dynamics in high-TB burden settings, where community transmission is pervasive, making it difficult to rely solely on household-related predictors to identify at-risk individuals [35–37]. In high-TB incidence settings, household-related risk factors alone do not sufficiently capture the multifaceted nature of *Mtb* exposure and infection from a single exposure, as prior transmission events inside or outside the household may have occurred [35]. The high prevalence of *Mtb* infection in such environments complicates the identification of new transmission events and makes it difficult to discriminate between individuals recently infected and those with prior exposure.

Additionally, the high prevalence of HIV in these settings may partly explain the poor performance of *Mtb* prediction models. Evidence suggests that HIV-related immunosuppression in people with TB can reduce the infectiousness of the individual [38]. This was reflected in our analysis, where HIV-positive index cases on ART were assigned the lowest risk scores. These findings further underscore the intricate interplay between community-level transmission dynamics and individual-level factors, such as HIV status and ART use, in shaping *Mtb* transmission patterns.

While both our models did not achieve good enough performance, this study represents an important step forward in understanding what is needed to provide targeted treatment of *Mtb* infection for adolescents and adults in high burden settings. In this context, prediction models cannot adequately discriminate between *Mtb* infected and non-infected adolescent and adults, as measured using IGRA. Also, given that IGRA is an imperfect reference standard with limited accuracy [39], the study might be predicting the wrong outcome especially when assessed at one time-point only. Therefore, a more nuanced understanding of *Mtb* transmission dynamics, recognizing the complex interplay between household-related factors and broader community-level influences is needed. Further collaborative, interdisciplinary innovation is essential to develop more accurate, easy-to-use, affordable and accessible tests that identify people with recent *Mtb* infection, those infected in the distant past and those not infected to inform targeted interventions for TB control and prevention.

Poor model performance may however also reflect the limitations of our study. For example, we only enrolled household contacts who were exposed to highly infectious TB index cases. Bacterial burden in the index case, most frequently categorized as smear positive and smear negative, is strongly associated with *Mtb* infection [40–42]. However, in this study TB index cases were only eligible if their sputum samples tested smear-positive or medium or high level Xpert MTB/Rif positive (which is equivalent to smear positive) meaning heterogeneity of bacterial burden in the index cases was limited. This limits our ability to generalize findings to lower bacterial burden categories (e.g., smear-negative or trace Xpert positive), which have also been shown to contribute to *Mtb* transmission.

In conclusion, our study provides valuable insights into the challenges of developing and or implementing predictive models for *Mtb* infection, ultimately aimed at identifying those most recently infected, in high burden settings characterized by intense community transmission. Use of individual and household predictors does not add significant benefit over the treat-all or treat-none strategies currently being the most implemented strategy in low resource settings.

## Supporting infromation

S1 Table Inclusion and Exclusion criteria for the ERASE-TB study.
(DOCX)

S2 Table List of candidate predictors and how data was collected.
(DOCX)

S3 Table Comprehensive model predictors.
(DOCX)

S1 Fig Receiver Operating Characteristic curve: Complete cases of baseline IGRA and last available follow-up IGRA results.
(DOCX)

S1 Code R code for sensitivity analysis using baseline complete cases.
(DOCX)

S2 Code R code for calibration plot of basic prediction model.
(DOCX)

S1 Checklist Implementation inclusivity questionnaire.
(DOCX)
