# Supplementary material for: Prediction models for Mtb infection among adolescent and adult household contacts in high tuberculosis incidence settings
Source: PLOS Glob Public Health. 2025 Mar 31;5(3):e0004340. doi: 10.1371/journal.pgph.0004340 (PMC11957366; doi:10.1371/journal.pgph.0004340)
Supplement: S1 Table — (DOCX) [file pgph.0004340.s001.docx]

**S1 Table: Inclusion and Exclusion criteria for the ERASE-TB study**

| TB Index Cases | Household Contacts |
| --- | --- |
| **Inclusion Criteria**   - Age ≥ 18 years - Diagnosed with active pulmonary TB within the last four weeks - Positive sputum smear, i.e. >+2 positivity by Ziehl-Neelsen stain or Auramine-O stain OR ≥+1 positivity on WHO symptom scale OR ≥medium positivity by GeneXpert (if no microscopy performed) - Less than seven days on anti-TB treatment since diagnosis - Able to spontaneously produce sputum - Living with at least another person aged ≥10 years in the same household with a firm unchanged address for six months - Written informed consent to conduct socio‐economic and clinical questionnaire, to provide a sputum sample for culture and sequencing, and to approach the household members. | **Inclusion Criteria**   - Age ≥ 10 years - Recent, substantial exposure to an infectious TB case in the household, defined as sleeping at least three nights per week in the same household in the last four weeks - Written informed consent for study participation, including HIV testing, and home visits by the study team for follow‐up (for minors <18 yr.: consent of the parent/guardian, assent of the participant) - If HIV negative: not on TB preventive therapy   **Exclusion Criteria**   - On current TB treatment for active TB, completed within the last 30 days. - Circumstances that raise doubt on free, uncoerced informed consent (e.g., in a mentally handicapped person) - Prisoners |
