# Supplementary material for: Prediction models for Mtb infection among adolescent and adult household contacts in high tuberculosis incidence settings
Source: PLOS Glob Public Health. 2025 Mar 31;5(3):e0004340. doi: 10.1371/journal.pgph.0004340 (PMC11957366; doi:10.1371/journal.pgph.0004340)
Supplement: S2 Table — (DOCX) [file pgph.0004340.s002.docx]

**S2 Table: List of candidate predictors and how data was collected**

| Predictor | Source | How it was interpreted |
| --- | --- | --- |
| Data from household contact | | |
| Household contact’s age | Household contact baseline questionnaire | Calculated from Date of birth in full years at date of enrolment |
| Household contact’s sex | Household contact baseline questionnaire | Asked directly from participants |
| Household contact’s HIV status | Household contact baseline questionnaire  HIV test results | Asked from participant at enrolment. Consenting participants are then tested within the study if reported negative or unknown. |
| Did the household contact care for the index case during illness | Household contact baseline questionnaire | Did you look after the household member with TB? *Yes/No* |
| Household contact’s nutritional status | Physical examination | BMI calculated from weight and height for all participants.  Categorized according to WHO guideline for adults and children |
| Data from the index case | | |
| Index case HIV status (incl. ART status) | Index case questionnaire | Asked directly from index case at enrolment |
| Index case symptom duration | Index case questionnaire | Asked directly from index case at enrolment |
| Index case strength of smear results | Index case questionnaire | Recorded from patient records at facility of diagnosis |
| Household variables | | |
| Household crowding | Composite variable | Calculated from the number of people in a household and number of rooms.  Crowding according to the UN (≥3 people per room) |
| Household food insecurity | Household contact baseline questionnaire | How many meals do you eat per day?  Food insecure if <3 meals |
| Household indoor smoking | Key informant questionnaire | Does anyone currently smoke tobacco indoors? *Yes/No* |
