# Supplementary material for: Prediction models for Mtb infection among adolescent and adult household contacts in high tuberculosis incidence settings
Source: PLOS Glob Public Health. 2025 Mar 31;5(3):e0004340. doi: 10.1371/journal.pgph.0004340 (PMC11957366; doi:10.1371/journal.pgph.0004340)
Supplement: S3 Table — (DOCX) [file pgph.0004340.s003.docx]

**S3 Table: Comprehensive model predictors**

| Predictors |  | Coefficient | P-Value |
| --- | --- | --- | --- |
| Sex | Female | Ref |  |
|  | Male | -0.011 | 0.93 |
| Age (years) | 10-14 | Ref |  |
|  | 15-17 | 0.298 | 0.20 |
|  | 18-25 | 1.295 | 0.28 |
|  | 26-35 | 1.483 | 0.22 |
|  | 36 + | 1.881 | 0.12 |
| HHC HIV status | Negative | Ref |  |
|  | Positive | -0.326 | 0.05 |
|  | Unknown | -0.388 | 0.46 |
| Cared for the index | No | Ref |  |
|  | Yes | 0.262 | 0.04 |
| BMI category | Healthy | Ref |  |
|  | Underweight | 0.343 | 0.76 |
|  | Overweight | 0.197 | 0.89 |
|  | Obese | 0.284 | 0.84 |
| Index HIV status | Negative | Ref |  |
|  | Positive, not on ART | -0.418 | 0.06 |
|  | Positive, on ART | 0.003 | 0.98 |
|  | Unknown | 0.377 | 0.08 |
| Index symptom duration | <1 month | Ref |  |
|  | 1≤ months<3 | 0.565 | <0.001 |
|  | 3 months and above | 0.850 | <0.001 |
| Household crowding (≥3 people per room) | No | Ref |  |
|  | Yes | 0.290 | 0.04 |
| Gene Xpert positivity for index case | Medium | Ref |  |
|  | High | 0.089 | 0.46 |
| Food insecurity | No | Ref |  |
|  | Yes | 0.138 | 0.31 |
| Indoor smoking | No | Ref |  |
|  | Yes | -0.012 | 0.95 |
| Footnotes: HHC = Household contact | | | |
