# Supplementary material for: Prediction models for Mtb infection among adolescent and adult household contacts in high tuberculosis incidence settings
Source: PLOS Glob Public Health. 2025 Mar 31;5(3):e0004340. doi: 10.1371/journal.pgph.0004340 (PMC11957366; doi:10.1371/journal.pgph.0004340)

**S1 Fig: Receiver Operating Characteristic curve only including valid IGRA results at baseline (red) and the last available follow-up IGRA results**
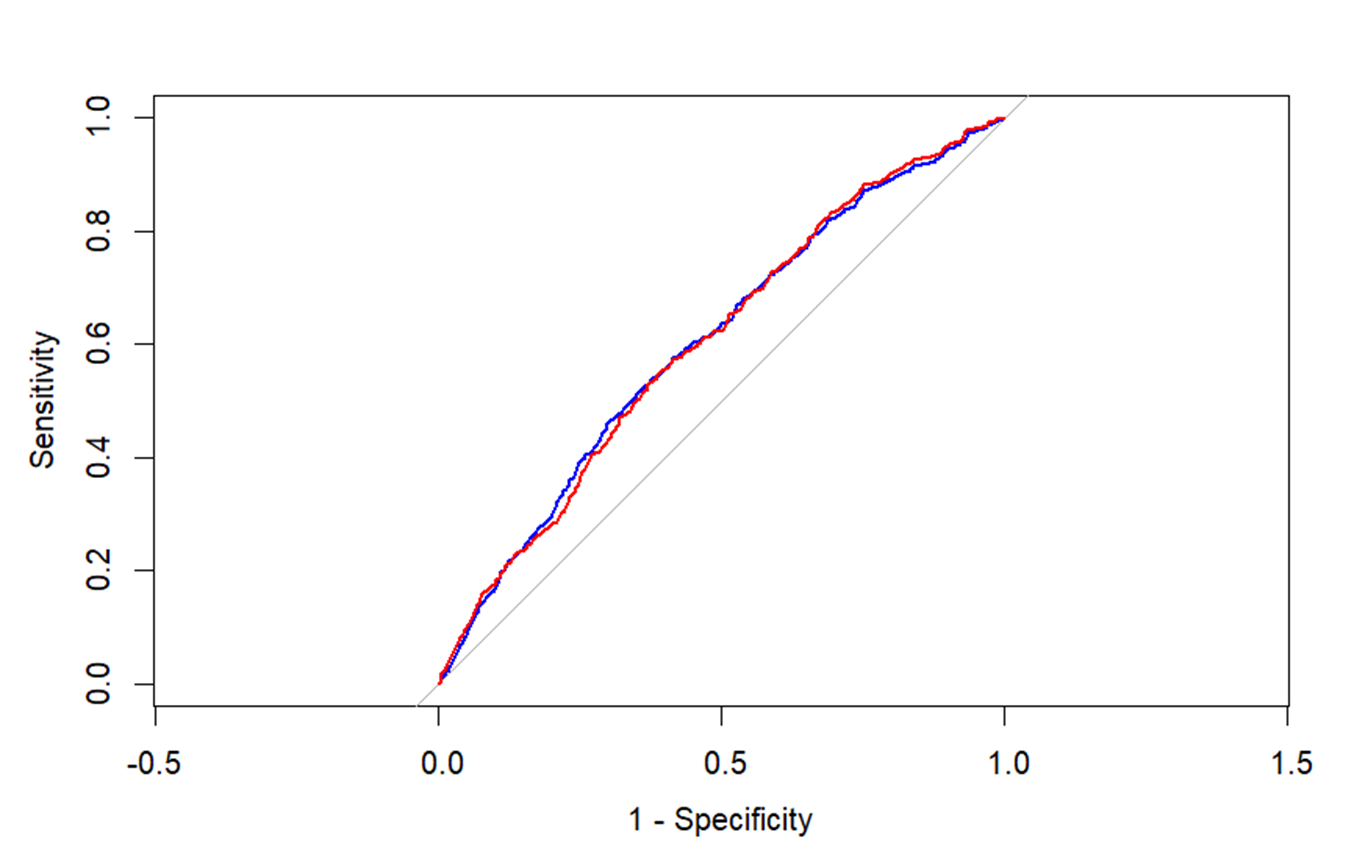

Supplement: S1 Fig — (DOCX) [file pgph.0004340.s004.docx]
