# Supplementary material for: Prediction models for Mtb infection among adolescent and adult household contacts in high tuberculosis incidence settings
Source: PLOS Glob Public Health. 2025 Mar 31;5(3):e0004340. doi: 10.1371/journal.pgph.0004340 (PMC11957366; doi:10.1371/journal.pgph.0004340)
Supplement: S1 Code — (DOCX) [file pgph.0004340.s005.docx]

**S1 Code: R Code for comprehensive model, basic model development and sensitivity analysis using baseline complete cases**

**####Comprehensive model**

ctrl <- trainControl(method = "cv", number = 10)

set.seed(123)

model_1b <- train(IGRA_Result_Overall ~ Sex +

Age +

`HIV status`+

`Nutritional status (adult)` +

`Food insecurity` +

`Cared for index?` +

`Index HIV status` +

`Index Symptom duration_Cat` +

`Gene xpert positivity for index`+

`Indoor smoking`+

`Household crowding according to UN (≥3 people per room)`, data = train, method = "glm", trControl = ctrl, na.action = na.pass)

**####Basic model**

model_basic <- train(IGRA_Result_Overall ~ Age +

`Cared for index?` +

`Index HIV status` +

`Index Symptom duration_Cat` +

`Gene xpert positivity for index`+

`Household crowding according to UN (≥3 people per room)`, data = HHC_IGRA, method = "glm", trControl = ctrl, na.action = na.pass)

**####Sensitivity analysis**

**###Using baseline IGRA**

HHC_IGRA_complete_cases <- HHC_IGRA %>%

filter(Q_602g_Ntb %in% c("IFN = Negative", "IFN = Positive"))

HHC_IGRA_complete_cases <- HHC_IGRA_complete_cases[complete.cases(HHC_IGRA_complete_cases[, vars_to_check2]), ]

# Predicting using the model on complete cases

predictions_model_basic <- predict(model_basic, newdata = HHC_IGRA_complete_cases, type = "raw")

predictions_model_basic_prob <- predict(model_basic, newdata = HHC_IGRA_complete_cases, type = "prob")

confusion_model_basic <- confusionMatrix(predictions_model_basic, HHC_IGRA_complete_cases$IGRA_Result_Overall)

predictions_model_basic_numeric <- as.numeric(predictions_model_basic)

predictions_model_basic_prob_numeric <- as.numeric(predictions_model_basic_prob[, 2])

roc_curve_model_basic <- roc(response = HHC_IGRA_complete_cases$IGRA_Result_Overall, predictor = predictions_model_basic_prob_numeric)

auc <- round(auc(roc_curve_model_basic), 2)

# Plot ROC Curve

plot(roc_curve_model_basic, main = "ROC Curve for '6-variable' basic model", col = "red", lwd = 2, legacy.axes = TRUE)

legend("bottomright", legend = paste("complete cases AUC =”, auc), col = "red", lwd = 2)

**###Using endline IGRA**

HHC_IGRA_V5 <- HHC_IGRA_V5[complete.cases(HHC_IGRA_V5[, vars_to_check3]), ]

predictions_model_HIV_excluded2 <- predict(model_HIV_excluded, newdata = HHC_IGRA_V5, type = "raw")

predictions_model_HIV_excluded2_prob <- predict(model_HIV_excluded, newdata = HHC_IGRA_V5, type = "prob")

confusion_model_HIV_excluded2 <- confusionMatrix(predictions_model_HIV_excluded2, HHC_IGRA_V5$IGRA_Result_Overall)

print(confusion_model_HIV_excluded2)

predictions_model_HIV_excluded2_numeric <- as.numeric(predictions_model_HIV_excluded2)

predictions_model_HIV_excluded2_prob_numeric <- as.numeric(predictions_model_HIV_excluded2_prob[, 2])

# Calculate ROC Curve for endline IGRA

roc_curve_model_HIV_excluded2 <- roc(response = HHC_IGRA_V5$IGRA_Result_Overall, predictor = predictions_model_HIV_excluded2_prob_numeric)

auc <- round(auc(roc_curve_model_HIV_excluded2), 2)

# Plot ROC Curve for endline

plot(roc_curve_model_HIV_excluded2, main = "Endline IGRA", col = "blue", lwd = 2, legacy.axes = TRUE)

# Add ROC Curve for complete cases baseline IGRA

plot(roc_curve_model_basic, col = "red", lwd = 2, add = TRUE, legacy.axes = TRUE)

legend_text <- c(

paste("Endline IGRA (AUC =", format(auc_a, digits = 3), ")", sep = ""),

paste("complete cases (AUC =", format(auc_b, digits = 3), ")", sep = "")

)

legend("bottomright", legend = legend_text, col = c("blue", "red"), lwd = 2, bty = "n")
