# Supplementary material for: Prediction models for Mtb infection among adolescent and adult household contacts in high tuberculosis incidence settings
Source: PLOS Glob Public Health. 2025 Mar 31;5(3):e0004340. doi: 10.1371/journal.pgph.0004340 (PMC11957366; doi:10.1371/journal.pgph.0004340)
Supplement: S2 Code — (DOCX) [file pgph.0004340.s006.docx]

**S2 Code: R code for calibration plot**

test$IGRA_Result_Overall <- ifelse(test$IGRA_Result_Overall == "IFN positive", 1,

ifelse(test$IGRA_Result_Overall == "IFN negative", 0, NA))

calibration_data <- data.frame(actual = test$IGRA_Result_Overall,

predicted_prob = predictions_model_HIV_excluded_prob)

calibration_data <- calibration_data %>%

mutate(bin = ntile(predicted_prob, 10))

calibration_summary <- calibration_data %>%

group_by(bin) %>%

summarize(mean_predicted_prob = mean(predicted_prob),

observed_proportion = mean(actual))

# Plot the calibration curve

ggplot(calibration_summary, aes(x = mean_predicted_prob, y = observed_proportion)) +

geom_line(color = "blue") +

geom_point(size = 2) +

geom_abline(slope = 1, intercept = 0, linetype = "dashed", color = "red") +

labs(title = "Calibration Plot",

x = "Mean Predicted Probability",

y = "Observed Proportion") +

theme_minimal()
